# Process Simulation and Economic Evaluation of the [bmim][FeCl_4_] Ionic Liquid for H_2_S Direct Conversion to Elemental Sulfur

**DOI:** 10.1021/acsomega.5c11851

**Published:** 2026-07-08

**Authors:** H. Abuzar Ahsan, M. Azmi Bustam, H. Ali Murtaza, Abid Mehmood, Bawadi Abdullah

**Affiliations:** † Department of Chemical Engineering, 61772 Universiti Teknologi PETRONAS, Bandar Seri Iskandar 32610, Perak, Malaysia; ‡ Centre of Carbon Capture, Utilization and Storage (CCCUS), Institute of Sustainable Energy and Resources (ISER), Universiti Teknologi PETRONAS, Bandar Seri Iskandar 32610, Perak, Malaysia; § Department of Chemical Engineering, King Faisal University, 36362 Al-Ahsa, Saudi Arabia

## Abstract

The urgent demand for cleaner industrial gas treatment and sustainable sulfur recovery has intensified the search for alternatives to conventional high-temperature processes. In this work, the ionic liquid transition metal (ILTM) process using [bmim]­[FeCl_4_] was designed and simulated in Aspen Plus (v14), while the conventional Claus process was modeled in Aspen HYSYS (v14). [bmim]­[FeCl_4_] was defined as a pseudocomponent, with thermophysical properties estimated using COSMO-RS and the Valderrama–Robles method. A refinery off-gas containing 91.4 mol % H_2_S served as the feed basis. Technical performance was assessed through mass and energy balances and sulfur recovery efficiency, while economic performance was evaluated in Aspen Process Economic Analyzer (APEA). Technically, the ILTM achieved a sulfur recovery efficiency of 99.68% under milder conditions. Its simplified single-stage absorption regeneration cycle eliminates catalytic reactors and high-temperature units, reducing the process complexity and energy demand. Economically, ILTM lowered capital expenditure (CAPEX) by 43.96% and operating expenditure (OPEX) by 38%, resulting in a total annualized cost (TAC) 42% lower than that of the conventional process. Overall, the [bmim]­[FeCl_4_]-based ILTM technology represents a promising low-carbon alternative to the Claus process, exhibiting zero direct CO_2_ emissions compared with approximately 0.78 t of CO_2_/t of S generated in the conventional process. However, further analysis is needed to reduce the ionic liquid cost and validate large-scale operation through pilot-scale studies.

## Graphical abstract

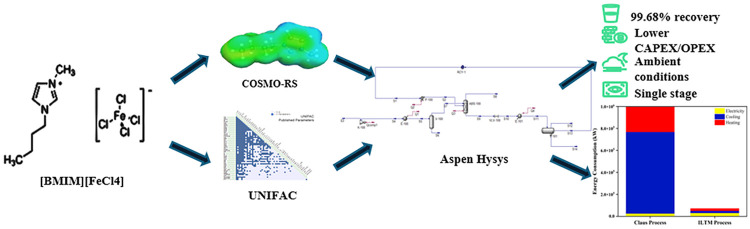

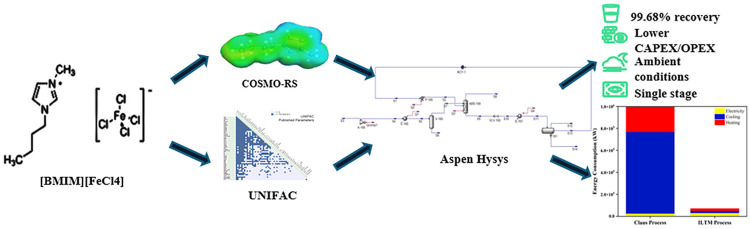

## 1 Introduction

The global demand for cleaner fuels with the least sulfur content has strengthened due to strict environmental regulations to limit sulfur dioxide emissions, a primary contributor to acid rain and pollution.^1^ Regulatory agencies worldwide have tightened emission limits for H_2_S and SO_2_ in both ambient air and industrial exhaust streams, and penalties for noncompliance have increased accordingly.^2^ Consequently, industries are under growing pressure to manage hydrogen sulfide (H_2_S), a toxic flammable gas with the distinct odor of rotten eggs. H_2_S is commonly produced as a byproduct during petroleum refining, natural gas processing, and petrochemical manufacturing.^3^ In refining operations, sulfur compounds in crude oil are converted into H_2_S during desulfurization, while in gas processing, H_2_S is removed to prevent corrosion and meet product quality standards.^4^ Beyond its environmental impact, H_2_S poses significant safety and economic challenges. When it reacts with metal surfaces, it forms brittle and porous iron sulfide (FeS) layers that deteriorate under fluctuating operation conditions, leading to localized corrosion and equipment damage.^5,6^ The financial consequences of corrosion-related losses in the global oil and gas industry are estimated at around USD 60 billion annually.^6^ As energy demand continues to grow, effective H_2_S removal remains essential to protect equipment, ensure safe operations, and comply with environmental regulations.^7,8^ Conventional technologies for removing or converting H_2_S, such as the Claus process, LO-CAT process, amine scrubbing, adsorption, and hydrodesulfurization (HDS), are well-established but face notable limitations.^4,9^ The Claus process and HDS are energy-intensive and less effective when treating gas streams containing low H_2_S concentrations.^10,11^ Similarly, amine scrubbing and adsorption systems can be costly to operate and may generate secondary waste streams that complicate removal.^12^ The LO-CAT process offers a milder and more environmentally friendly approach but struggles with catalyst degradation and byproduct management.^13^ These shortcomings underscore the need for alternative desulfurization technologies that are not only cleaner and more efficient but also economically sustainable. Table 1 presents a comparative assessment of established H_2_S removal technologies, alongside the ILTM-based process investigated in this study. The comparison highlights key differences in operating conditions, sulfur removal efficiency, energy consumption, product and byproduct formation, and capital and operating cost characteristics. This side-by-side evaluation provides context for positioning the ILTM approach relative to conventional desulfurization routes and clarifies its potential advantages and limitations.

**Table 1:** Comparison of Conventional and ILTM-Based H_2_S Removal Processes

technology	physicochemical principle	operating conditions	H_2_S removal efficiency (%)	main product/byproduct	CAPEX (M USD)	OPEX (M USD yr^–1^)	advantages	limitations	refs
Claus process	Thermal oxidation and catalytic conversion	Thermal stage: 1000–1200 °C, catalytic stages: 200–400 °C	95–99	Elemental sulfur; tail-gas SO_2_	57.35	2.45	Industrially mature, high throughput, and robust operation	High fuel consumption, inefficient at low H_2_S concentrations and requires tail-gas treatment	^14^
LO-CAT Process	Aqueous iron chelate redox cycling	Ambient–moderate temperature; atmospheric pressure	95–98	Elemental sulfur; degraded chelate waste	1.12–1.33	0.036–0.039	Effective at low H_2_S partial pressures and mild conditions	Chelate degradation, wastewater generation, and chemical makeup required	^15^
Amine scrubbing	Physical/chemical absorption–desorption	Absorption: 40–60 °C; regeneration: 100–120 °C	85–99	Acid gas stream (H_2_S-rich)	64.56–110.72	2–2.5	Well-established and flexible for varying feeds	High regeneration energy and secondary sulfur recovery required	^16^
Adsorption	Chemisorption/physisorption	Ambient temperature; fixed-bed operation	80–99 (bed-dependent)	Spent sulfided sorbent	0.205–1	(3.9–4.6) × 10^–5^	Low-temperature and modular design	Frequent sorbent replacement and solid waste disposal	^17^
Hydrodesulfurization (HDS)	Catalytic hydrogenation	300–400 °C; high H_2_ pressure	90–99	Hydrocarbons + H_2_S	31–42	0.5–0.8	Highly effective for organosulfur removal	High hydrogen demand and unsuitable for dilute gas streams	^11,18^
ILTM-based Process	Nonaqueous ionic liquid redox oxidation	Ambient temperature-atmospheric pressure	∼99 (simulated)	Elemental sulfur dispersed in IL	32.13	1.53	Low-temperature operation, high selectivity, and compact equipment	Ionic liquid cost, sulfur separation, and IL handling	This work

In recent years, ionic liquids (ILs) have emerged as promising candidates for gas treatment applications because of their unique physicochemical properties, such as negligible vapor pressure, high thermal stability, and tunable solvation capacity.^19–21^ Among them, the magnetic ionic liquid 1-butyl-3-methylimidazolium tetrachloroferrate ([bmim]­[FeCl_4_]) has drawn significant interest. First introduced by Hayashi and Hamaguchi, [bmim]­[FeCl_4_] is distinguished by the dual functionality of its [FeCl_4_]^−^ anion, which exhibits both paramagnetic and redox-active properties.^22,23^ These features enable it to act simultaneously as an absorbent and a reactive medium, efficiently capturing H_2_S and converting it into elemental sulfur under mild operating conditions.^23,24^ The magnetic nature of [bmim]­[FeCl_4_] further enhances its practicality, allowing for straightforward separation and recovery under an external magnetic field.^25^ This simplifies downstream purification, minimizes solvent loss, and enables solvent reuse, thereby reducing both energy consumption and process costs.^26^

Beyond its desulfurization potential, [bmim]­[FeCl_4_] has demonstrated versatility as a catalyst and solvent in various chemical processes. For instance, it has been successfully applied in oleic acid esterification, achieving high conversion efficiency with excellent recyclability.^27^ Its thermal stability, low viscosity, and broad liquid range make it particularly attractive for industrial applications.^25,28,29^ Moreover, the Lewis acidity of the [FeCl_4_]^−^ anion enhances its selective extraction capacity toward sulfur- and nitrogen-containing compounds, improving its desulfurization performance in complex petroleum matrices.^28,30,31^ These properties collectively position [bmim]­[FeCl_4_] as an effective and environmentally friendly solvent for sulfur removal and conversion.

Recent investigations support the growing promise of IL-based systems for H_2_S capture and sulfur recovery. Pilot-scale studies have reported sulfur recovery rates exceeding 98–99%, with markedly lower energy demands compared to the traditional Claus process.^32,33^ Such findings highlight the technical feasibility of these systems; however, the high production cost of ionic liquids remains a key barrier to widespread industrial adoption.^34^ As a result, further research is needed to optimize formulations and to evaluate their economic competitiveness on a larger scale. While previous studies have focused primarily on the chemical behavior and reaction mechanisms of ionic liquid transition metal (ILTM) systems, there is still limited understanding of their process-level integration and techno-economic performance compared to established sulfur recovery technologies.^35^

To address this gap, the present study investigates the technical and economic performance of an ILTM-based process for H_2_S conversion to elemental sulfur using [bmim]­[FeCl_4_] as a model solvent.^36^ The process is designed and simulated using Aspen Plus and benchmarked against the conventional Claus process to assess its industrial viability. A comprehensive techno-economic analysis (TEA) is conducted to evaluate sulfur recovery efficiency, energy and utility requirements, solvent costs, and overall economic performance.^37–39^ By directly comparing ILTM-based sulfur recovery to the Claus process, this work provides actionable insights for industries seeking cleaner, more energy-efficient, and cost-effective desulfurization solutions. The results demonstrate that ILTM-based technologies that leverage the multifunctional and recyclable nature of [bmim]­[FeCl4] can achieve high H_2_S conversion under milder operating conditions, while potentially reducing both capital and operating expenses. Ultimately, this research highlights the potential of ILTM-based processes as viable, sustainable alternatives for industrial gas treatment, contributing to greener and more economically resilient energy systems.^40^

## 2 Methods

### 2.1 Process Modeling

The ILTM-based process for direct H_2_S conversion was simulated and compared with the conventional Claus process using Aspen Plus (v14) and Aspen HYSYS (v14). These commercial simulators were selected due to their strong capabilities in handling both conventional and nonconventional components. Since the ionic liquid [bmim]­[FeCl_4_] was not available in the Aspen Plus component database, it was defined as a pseudocomponent. Property estimation was performed using a COSMO-RS-based approach that integrates quantum chemical calculations and thermodynamic predictions. Fundamental molecular parameters, including molecular weight (MW) and boiling point (BP), were first determined using quantum chemical methods and then processed in COSMOthermX to predict physical and thermodynamic properties shown in Table 2.^41,42^ To ensure the reliability of simulation predictions, the ionic liquid [bmim]­[FeCl_4_] was modeled as a pseudocomponent in Aspen Plus using experimentally validated thermophysical properties. Density, viscosity, heat capacity, and thermal conductivity values were sourced from literature data available.^30,43,44^ Model validation against experimental data showed deviations of less than 3% for density and specific heat and within ±5% for viscosity at 298 K, confirming that the pseudocomponent representation provides a reliable basis for process simulation. The Valderrama–Robles group contribution method (Table 3) was applied to estimate the critical properties of [bmim]­[FeCl_4_], with its full thermophysical definition in Aspen Plus. The UNIFAC property package was used as the thermodynamic package for the ILTM process, as it was extensively applied for IL systems due to its accuracy in representing nonideal mixtures.^45^ This modeling framework enabled reliable prediction of H_2_S-IL interactions under industrially relevant operating conditions.^46,47^ The process flow diagram for the ILTM-based process is depicted in Figure 1.

**Figure 1:**
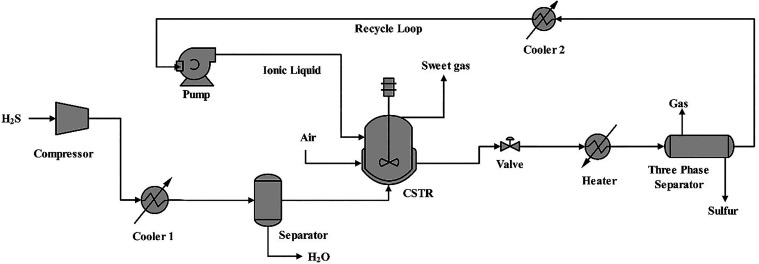
Process flow diagram for the ILTM process.

**Table 2:** Estimated Physical Parameters of [bmim]­[FeCl_4_] at 25°C

IL	MW (g/mol)	ρ (g/cm^3^)	μ (cP)	M.P (°C)	B.P (°C)	ΔHR (kJ/mol)
[Bmim][FeCl_4_]	336.9	1.322	26.81	18.36	508.22	–417

**Table 3:** Valderrama–Robles Equations

parameter	equations
Boiling Point	Tb(K)=198.2+∑nΔTb
Critical Pressure	Pc(bar)=MW/(C+∑nΔPc)2
Critical Temperature	Tc(K)=Tb/A+B∑nΔTc−(∑nΔTc)2
Compressibility Factor	Zc=PcVc/RTc
Critical Volume	$\mathrm{Vc}\;\left({\mathrm{cm}}^{3}/\mathrm{mol}\right)=D+\sum n\Delta \mathrm{Vc}$
Acentric Factor	ω=(Tb−43)(Tc−43)/(Tc−Tb)(0.7Tc−43)log(Pc/Pb)−(Tc−43)/(Tc−Tb)log(Pc/Pb)+log(Pc/Pb)−1

### 2.2 Process Simulation Configuration

To ensure consistency, both the ILTM and Claus processes were simulated under identical inlet gas conditions representing a typical Malaysian oil refinery (Table 4).^48^ The ILTM process flowsheet consisted of a compressor, cooler, reactor, three-phase separator, heater, and recycle loop. This configuration enabled efficient H_2_S conversion, moisture removal, and IL regeneration. The H_2_S oxidation in the ILTM reactor was modeled using a kinetic approach rather than assuming chemical equilibrium. A pseudohomogeneous kinetic model was implemented in Aspen Plus, reflecting the liquid-phase redox cycle between Fe^3+^ and Fe^2+^ species in the [FeCl_4_]^−^ anion. This choice was based on experimental evidence reported in the literature showing that iron­(III)-based ionic liquids, including [bmim]­[FeCl_4_], promote rapid redox cycling between Fe^3+^ and Fe^2+^ species. The reduced Fe^2+^ is efficiently regenerated to Fe^3+^ through oxidation by oxygen. As a result, near-complete H_2_S conversion was achieved under mild operating conditions and short contact times.^49,50^ A first-order dependence on both H_2_S and Fe^3+^ concentrations was assumed, with gas–liquid mass transfer considered nonlimiting under the simulated operating conditions. The overall reaction considered in the model was the selective oxidation of H_2_S to elemental sulfur, as shown in eq 1.

1 $$H_{2}S+\frac{1}{2}O_{2}\to S+H_{2}O$$

**Table 4:** Inlet Gas Stream Characteristics

parameters	value
Temperature (°C)	177
Pressure (kg/cm^2^g)	0.89
Molar Flow rate (kmol/h)	256.236

component	amine regeneration off-gas (mole %)
H_2_S	91.4
NH_3_	0.26
CO_2_	Traces
COS	Traces
H_2_	2.43
Water Vapor	4.75
Hydrocarbons	
C1	0.0033
C2	0.0008
C3	0.0007
iC4	0.0028
nC4	0.0034
C5+	0.0006

The [bmim]­[FeCl_4_] was treated as a catalytic medium, and its concentration was assumed to remain constant due to continuous regeneration. Side reactions leading to SO_2_ or sulfate formation were neglected, as previous studies have shown that iron-based ionic liquids favor elemental sulfur formation with high selectivity at ambient temperature and low oxygen partial pressures.^51,52^ Sensitivity analysis demonstrated that a ±20% variation in the rate constant altered overall H_2_S conversion by less than 3%, confirming that the simulation outcomes are only moderately sensitive to the chosen kinetic parameters (Figure S1). This validates the appropriateness of the kinetic model and supports the reliability of the predicted reactor performance. Heat duties for the ILTM process units were determined automatically in Aspen Plus v14 through full energy balance calculations using the UNIFAC property method. UNIFAC was selected due to its proven capability in predicting phase equilibria and excess enthalpies for IL systems reported in previous studies.^53–56^ All reactor and separation unit energy requirements, including IL regeneration, were based on this thermodynamic framework. Alternative models (e.g., NRTL, ELECNRTL) were not simulated because these property models require complete interaction parameter sets, which are unavailable for [bmim]­[FeCl_4_].

The Claus process was simulated in Aspen Hysys (v14) using a Sulsim property package. The process included a reaction furnace, multiple catalytic reactors, condensers, and an incinerator.^57^ The thermal furnace was modeled using an equilibrium reactor (RGibbs) block, assuming complete combustion and thermal decomposition of H_2_S and hydrocarbons to achieve thermodynamic equilibrium at high temperatures (>1250 K). This approach is well-established for representing the fast, high-temperature reactions in the thermal stage of the Claus process.^57^ In contrast, the downstream catalytic reactors were modeled using kinetic reactor (RPlug) blocks, incorporating first-order reaction kinetics for the Claus and tail-gas conversion steps based on experimentally validated rate expressions reported by Amer et al. and Zahid et al.^58,59^ Using this hybrid equilibrium–kinetic configuration captures both the rapid thermal equilibration in the furnace and the rate-limited behavior in catalytic zones. Sensitivity analysis indicated that using purely equilibrium models for all stages overestimates sulfur recovery by approximately 3–5%, while the hybrid kinetic–equilibrium model yields more realistic recovery values consistent with industrial performance benchmarks (94–97% sulfur recovery). This confirms that the chosen modeling framework accurately represents the process behavior under the specified operating conditions. Both models were developed to replicate industrially relevant conditions and to allow direct comparison of performance and operability.^60^

### 2.3 Technical Performance Evaluation

To evaluate sulfur recovery performance, both systems were simulated using a feed stream containing 234.2 kmol/h H_2_S, which falls within the typical industrial range of 100–400 kmol/h.^57^ In the ILTM process, H_2_S conversion and IL regeneration were modeled as a single-stage operation, while the Claus process was analyzed across multiple reaction and condensation stages to determine cumulative recovery. Sulfur recovery efficiency was calculated using eq 2

2 $$\mathrm{Sulfur recovered}\left(\%\right)=\frac{\mathrm{liquid sulfur produced}}{\mathrm{sulfur compounds in feed}}\times 100$$

This approach supported a direct comparison of H_2_S conversion and sulfur yield, thereby assessing the technical feasibility of the ILTM process compared to the Claus process. To ensure model reliability of the ILTM process simulation, convergence analysis was performed for the recycle loop, phase separator, and reactor block in Aspen Plus. The ILTM process flowsheet contains a vapor–liquid–solid separation stage as well as a redox reactor with internal recycles. Convergence was achieved using the Wegstein and Broyden algorithms with tight tolerances (1 × 10^–5^) for material and energy balances. Multiple initial guesses were tested to verify that the simulation converged to a physically consistent solution independent of starting conditions. The phase separators were validated by comparing predicted phase splits and compositions with experimental data for [bmim]­[FeCl_4_]-H_2_S systems reported in the literature.^61^ The reactor block also demonstrated stable convergence across a range of inlet H_2_S flow rates (0.5–3 kmol/h) and operating temperatures (298–333 K). The detailed sensitivity plots for H_2_S conversion across concentration and temperature ranges are provided in the supplementary data (Figures S2 and S3). These results confirm that the model is numerically robust and that the convergence behavior of the recycle loops and reaction sections does not influence the overall process predictions.

### 2.4 Techno-Economic Analysis

A comprehensive techno-economic analysis (TEA) was conducted to evaluate the capital and operational expenditures of the ILTM process. Equipment sizing, selection, and installation cost estimation were performed using Aspen Process Economic Analyzer (APEA) integrated with Aspen Plus (v14), employing the US_IP template and 2022 cost database.^62,63^ The fixed capital cost comprised the capital direct cost (CDC) and capital indirect cost (CIC), as shown in eq 3. Direct costs included equipment, piping, civil works, instrumentation, and labor for installation, while indirect costs covered engineering, construction management, contingency, and ionic liquid or catalyst allowances. Table 6 exhibits the details of CAPEX and OPEX. The working capital cost (WCC) was set at 10% of the fixed capital cost (FCC) (eq 4), and the total CAPEX was determined as eq 5

3 FCC=CDC+CIC

4 WCC=10%ofFCC

5 Total CAPEX=FCC+WCC

Operational expenditure (OPEX) was divided into variable and fixed components. Variable costs (VOC) included utilities such as electricity, steam, cooling water, and fuel gas, while fixed operating costs (FOC) covered maintenance, labor, plant overhead, and administrative expenses.^64,65^ Utility prices were taken from the default values in APEA and material cost data were sourced from the updated Sigma-Aldrich online database and input into APEA, where final cost estimates were automatically generated based on the specified process conditions.^66^ In this study, the assumed costs for the raw material, electrical and thermal utilities used in the OPEX calculation are summarized in Table 5. Equations 6–8 were used to calculate the total OPEX (Table 6).

6 TotalOPEX=VOC+FOC

7 $$\mathrm{VOC}=C_{\mathrm{utilities}}+C_{\mathrm{raw materials}}$$

8 $$\mathrm{FOC}=C_{\mathrm{labor}}+C_{\mathrm{maintenance}}+C_{\mathrm{overhead}}+C_{\mathrm{operating}}$$

**Table 5:** Raw Materials and Utility Cost

raw material/utilities	unit	value	refs
Al_2_O_3_	USD/kg	2	^67^
[bmim][FeCl4]	USD/kg	194	^67^
Electricity	USD/kWh	0.061	^68^
LP Steam	USD/kJ	0.0072	^69^
Steam @ 400 psi	USD/kg	0.0053	^69^
Fuel Gas	USD/kWh	0.07	^68^
Cooling Water	USD/kg	0.00003	^69^

**Table 6:** Basis of Capital and Operational Cost Estimation

CAPEX	OPEX
Plant general equipment	Electricity
Process equipment (columns, heat exchangers, pumps, and compressors),	Steam
Piping, steel, civil	Cooling water
Instrumentation, electrical, insulation, paint	Fuel gas
**Capital direct cost (CDC) (sum of the above)**	**Variable operating cost (VOC) (sum of the above)**
Engineering, design, and procurement cost	Maintenance (2% of FCC)
Other project costs: construction, taxes, contract fee, etc.	Operating Labor (20$/h) & Supervision (35$/h)
General and administrative overheads, etc.	Plant overhead (50% of M + OL + S)
Contingency and ionic liquid/catalyst cost	Operating charges (25% of OL + S)
**Capital indirect cost (CIC) (sum of the above)**	**Fixed operating cost (FOC) (sum of the above)**

The total annualized cost (TAC) was calculated using eqs 7 and 8

9 $$\mathrm{TAC}=\mathrm{CRF}\cdot \sum_{n}^{\mathrm{units}}{\mathrm{CAPEX}}_{n}+\sum_{m}{\mathrm{OPEX}}_{m}$$

10 $$\mathrm{CRF}=\frac{{i\cdot \left(1+i\right)}^{a}}{{\left(1+i\right)}^{a}-1}$$

where *i* is the interest rate and *a* is the annuity period. In this study, an interest rate of 8% and an annuity period of 20 years were assumed for capital cost annualization.^70^ A sensitivity analysis was performed to assess the influence of IL price variation within the range of 50–400 USD/kg because their long-term manufacturing costs remain unpredictable.^71^ The base-case IL price of 194 USD/kg for [bmim]­[FeCl_4_] was used to estimate the TEA.

### 2.5 Comparative Framework and Assumptions

To ensure a fair and impartial comparison, both processes were simulated under standardized operating parameters, including feed composition, temperature, pressure, and gas flow rate, as mentioned in Table 4. All financial and economic assumptions were clearly defined and applied consistently to both the ILTM and the Claus processes. The same project lifetime, financial structure, and macroeconomic conditions were assumed for each case to enable a fair and unbiased comparison, ensuring that the observed differences arise primarily from the process design and energy requirements rather than financial factors. The general TEA assumptions for both processes are presented in Table 7, and air is considered freely available. These assumptions ensured uniformity in the technical and economic evaluations. Sulfur recovery values obtained from simulation results were used to calculate process efficiency using eq 2. This framework provided a consistent and robust basis for comparing the technical feasibility, cost performance, and overall viability of the ILTM-based and Claus sulfur recovery systems.

**Table 7:** TEA General Assumptions

parameter	units	base value (assumed)
Plant lifetime	years	20
Annual operating hours	hr/year	8000
Discount rate	%	8
Construction period	years	2
Inflation	%/yr	2
Debt: Equity	%	60:40
Loan interest rate	%	8
Corporate tax rate	%	25

## 3 Results and Discussion

In this section, the outcomes of the process simulations and TEA conducted for the ILTM-based sulfur recovery process and the conventional Claus process are presented. The findings are organized into technical and economic evaluations to provide a clear comparison of performance, efficiency, and cost-effectiveness under identical operating conditions.

### 3.1 Technical Evaluation of Sulfur Recovery Processes

Both simulations were performed with a feed gas stream comprising 234.2 kmol/h H_2_S, consistent with typical refinery operating ranges and previous simulation studies.^57,72^ The sulfur recovery efficiency was determined from the material balance data extracted from the simulation results.

#### 3.1.1 ILTM Process

The simulation of the ILTM process was performed using Aspen Plus due to its reliability in modeling IL systems.^45,73,74^ The ionic liquid [bmim]­[FeCl_4_] was not included in the Aspen Plus database. It was defined as a pseudocomponent using the COSMO-RS and Valderrama–Robles methods. The resulting thermophysical parameters (Table 8) were incorporated into the model using the UNIFAC property package. The thermophysical properties assigned to the ionic liquid pseudocomponent were validated using reported experimental data for metal-containing imidazolium ionic liquids with similar structures. Specifically, viscosity, density, and heat capacity values for [bmim]­[FeCl_4_] and related chloro-ferrate ILs reported by Cruz et al. and Javid Safarov et al. were compared with the temperature-dependent correlations implemented in Aspen Plus.^30,36^ The simulated density and viscosity trends matched the experimental ranges reported for metal chloride ILs within less than 5% deviation. UNIFAC was selected because it is one of the few thermodynamic models in Aspen Plus capable of handling nonideal liquid mixtures containing ILs and polar gases.^45^ Prior studies have demonstrated that group-contribution models such as UNIFAC reasonably predict activity coefficients and vapor–liquid equilibria for imidazolium IL systems.^53–56^ Alternative models such as NRTL/ELECNRTL/UNIQUAC require complete interaction parameter sets, which are unavailable for metal-containing ILs, such as [bmim]­[FeCl_4_]. Attempts to estimate missing parameters resulted in nonconvergence, consistent with limitations discussed by Hossain et al. and Farajnezhad et al.^75,76^

**Table 8:** Scalar Properties of [bmim]­[FeCl_4_]

parameter	value	units
Normal boiling point (Tb)	672.42	K
Molecular weight (MW)	336.87	g/mol
Acentric factor (OMEGA)	1.31	-
Critical temperature (Tc)	848.81	K
Critical pressure (Pc)	25.38	Bar
Compressibility factor (Zc)	0.27	-
Critical volume (Vc)	755.00	Cc/mol

As shown in Figure 2, the feed gas was compressed from 1.89 to 2 bar and cooled to 30 °C to condense and remove moisture. The slight increase in pressure helps create suitable conditions for the reactions and removal of moisture, improving the purity of the recovered elemental sulfur.^77^ Then, the treated gas entered the reactor, where it contacted the pressurized IL and an equimolar oxygen stream. H_2_S was oxidized to elemental sulfur, and to enhance the purity of elemental sulfur and recover leftover hydrocarbons, the process involves lowering the pressure to 1 bar, while simultaneously increasing the temperature to 100 °C. The heater provides the necessary heating for water removal and IL regeneration. This reboiler type was selected due to the high viscosity of the ionic liquids.^78^ The outlet stream was directed to a three-phase separator producing phases of regenerated ionic liquid, elemental sulfur, and light gases. The three-phase separator assumes gas–liquid–solid equilibrium, in which sulfur precipitates as a separate solid phase according to phase-equilibrium predictions. This approach follows similar IL-based sulfur separation methodologies reported by Wang et al. and Gang Liu.^79,80^ Nonideal interactions were accounted for using UNIFAC activity coefficients. Entrainment and microemulsions are known challenges in IL systems; however, previous experimental studies show that metal-containing ILs exhibit strong sulfur–IL immiscibility,^81^ making complete phase disengagement a reasonable assumption.

**Figure 2:**
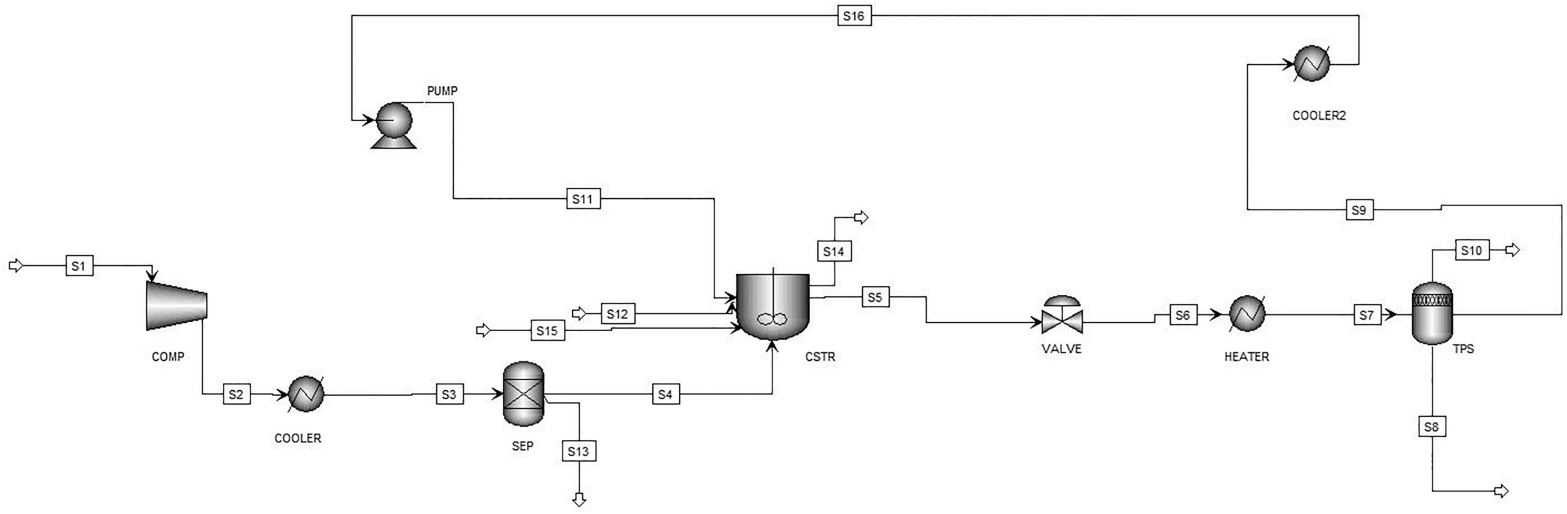
Simulation of the proposed ILTM process.

The ionic liquid was recycled to minimize the solvent loss and improve process sustainability. The moisture limits the performance of chlorometallate ionic liquids such as [Bmim]­[FeCl_4_] because water hydrolyzes the [FeCl_4_]^−^ anion, forming Fe­(OH)_*x*_ species and releasing HCl. This reaction disrupts the Fe^3+^ coordination environment, decreases the concentration of active oxidative sites, and alters IL properties including viscosity, acidity, and redox behavior.^50^ Mitigating water ingress was therefore essential. A heater was installed downstream of the catalytic reactor to remove water from the liquid stream. Overall, oxidative absorption of H_2_S was feasible for water contents below 80 vol % without operational obstruction.^82^ Other operational limitations include viscosity buildup during sulfur loading, interfacial fouling, and gradual loss of oxidative capacity, emphasizing the need for continuous regeneration and controlled residence times.^83^

The ILTM process achieved a sulfur recovery of 99.68%, corresponding to 234.18 kmol/h liquid sulfur from 234.20 kmol/h H_2_S. Similar results were reported by Nor Fariza et al. and Huanong Cheng et al., confirming the effectiveness of the same ionic liquid for H_2_S absorption.^51,84^

#### 3.1.2 Claus Process

The Claus process was simulated using Aspen HYSYS with the Sulsim property package employing the most common process flow configuration.^57^ The acid gas feed and wet air were mixed through a saturator before entering the furnace, where the partial combustion of H_2_S produced SO_2_ and H_2_O. The acid gas feed for the simulation was set on the basis of the parameters detailed in Table 3. The gas was cooled in a waste heat boiler and then passed through catalytic converters and condensers to produce elemental sulfur (Figure 3). In these reactors, the remaining H_2_S reacts with SO_2_ over a catalyst, typically alumina or titanium dioxide, to produce elemental sulfur. The tail gas, still containing minor amounts of unreacted H_2_S and SO_2_, was incinerated to oxidize residual sulfur species, reducing environmental emissions.

**Figure 3:**
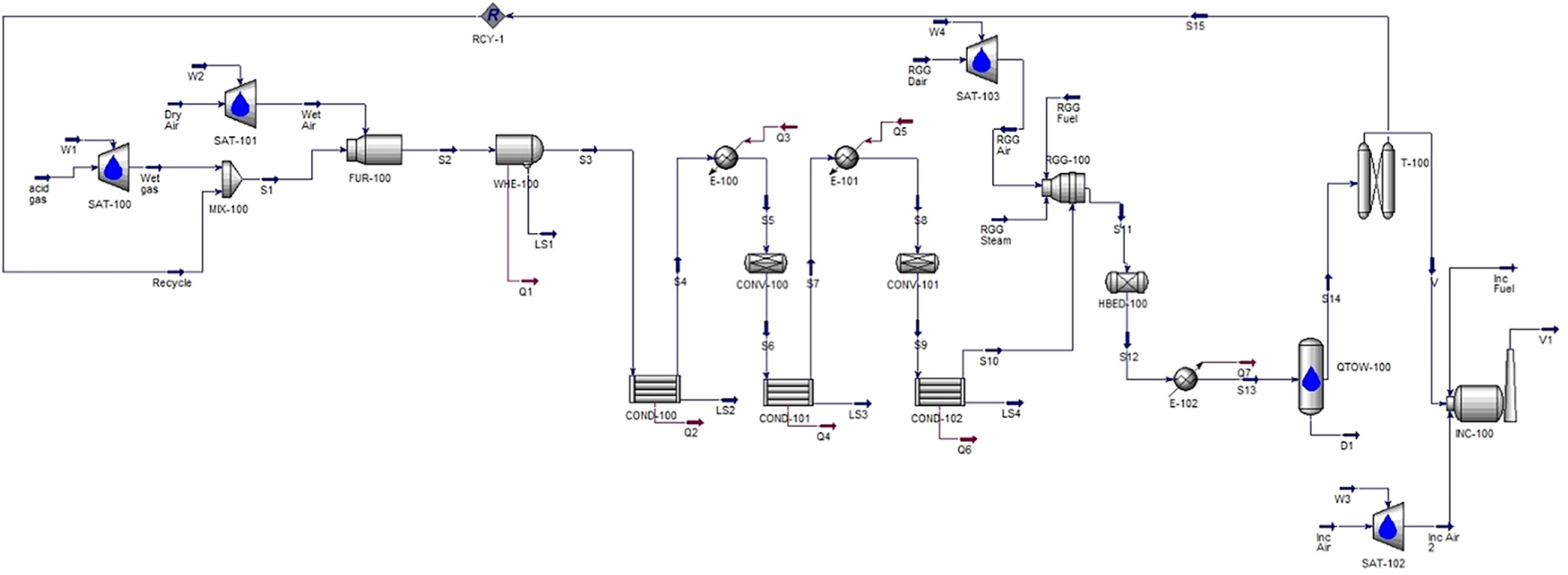
Simulation flowsheet for the Claus process.

A sensitivity analysis confirmed that increasing the H_2_S concentration in the feed leads to an increase in both the conversion rate and furnace temperature, consistent with the findings of Reza et al., as shown in Figure 4.^72^ Therefore, it can be concluded that the conducted simulations were precise. In the Claus process, the overall sulfur recovery was determined using eq 3, 99.56%, with multistage condensation and catalytic conversion illustrated in Figure 5.

**Figure 4:**
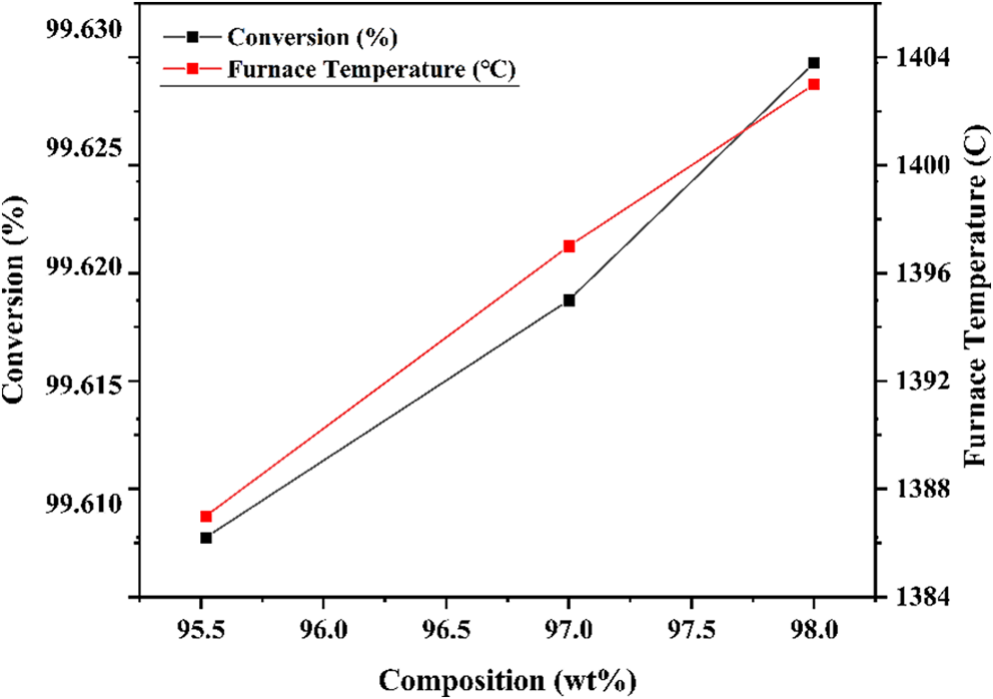
Sensitivity analysis of the Claus process.

**Figure 5:**
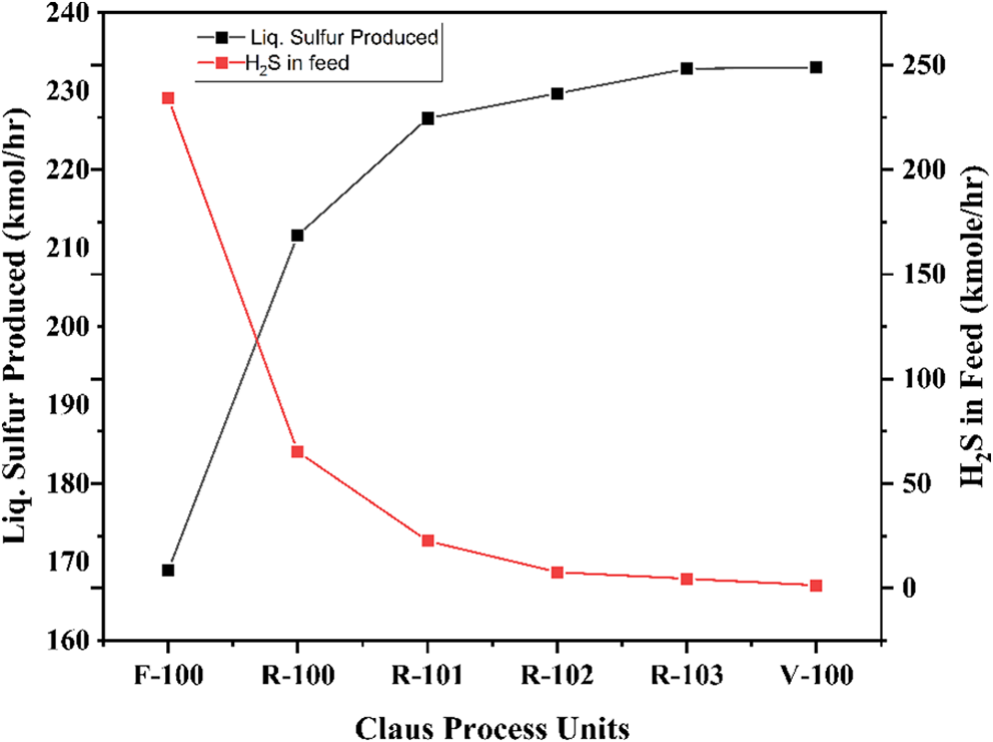
Production of liquid sulfur at each stage of the Claus Process.

Overall, both technologies achieved high sulfur recovery under identical conditions. The ILTM process slightly outperformed the Claus process under the milder operating conditions. The Aspen HYSYS results show that the conventional Claus process emits about 0.98 t of CO_2_/t of sulfur due to combustion in the thermal stage, whereas the ILTM process produces no direct CO_2_ emissions because it operates through redox cycling rather than fuel oxidation. This confirms the ILTM route as a lower carbon alternative for industrial desulfurization. The simulation results indicate that the ILTM process offers superior efficiency and operational simplicity, highlighting its potential as an environmentally and economically viable alternative for sulfur recovery.^85^

### 3.2 Economic Evaluation of Sulfur Recovery Processes

The techno-economic comparison revealed notable cost advantages for the ILTM process. The Claus process required a CAPEX of 57.35 million USD.^14^ In contrast, the ILTM process requires only 32.13 MUSD, which is 43.9% lower than that of the conventional process. This reduction is primarily due to the absence of high-temperature furnaces, multiple reactors, and corrosion-resistant equipment. OPEX showed a similar trend, with the ILTM process incurring 1.53 MUSD/year compared to 2.45 MUSD/year for the Claus process.^14^ The IL regeneration heat duty, determined by using the UNIFAC property method, was 1.1 GJ/ton of recovered sulfur. The 67.6% reduction results from lower energy consumption for cooling, heating, and compression are shown in Figure 6. This is primarily due to its operation at ambient temperatures, which significantly reduces the consumption of utilities, such as electricity, steam, and cooling water. The ability to reuse and regenerate the ILs further decreases long-term OPEX.^73,84,86^ The details of the CAPEX and OPEX calculations by APEA for the ILTM-based process are listed in Table 9.

**Figure 6:**
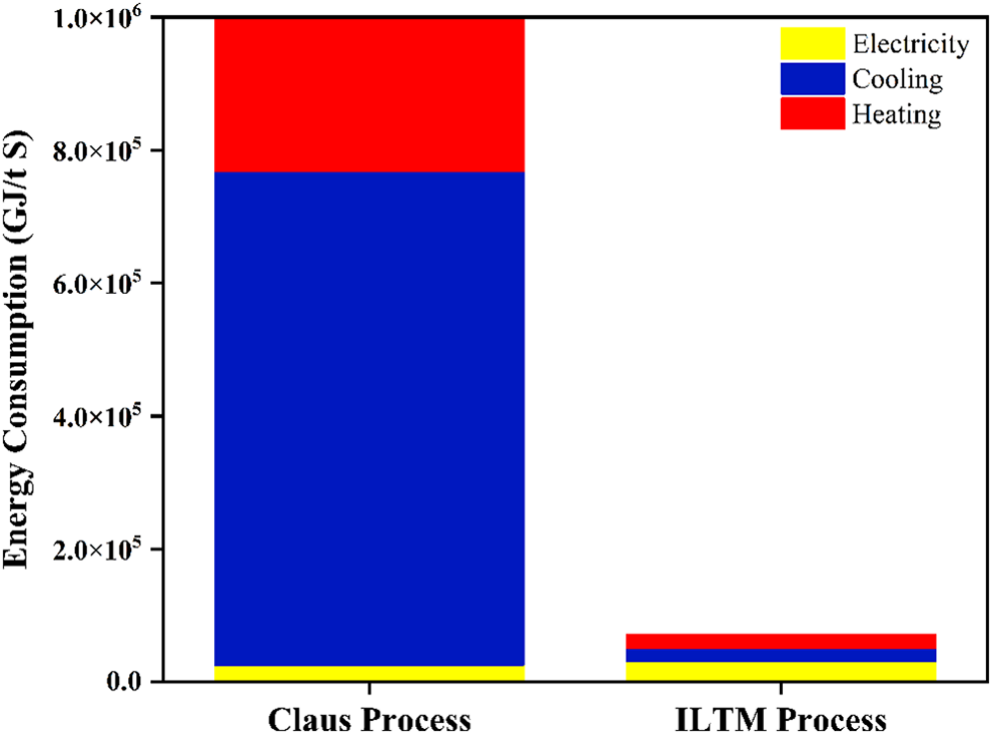
Energy consumption comparison.

**Table 9:** ILTM Process Capital and Operating Cost Calculated by APEA

summary	
Total capital cost [USD]	2,521,220
Total operating cost [USD/Year]	31,641,000
Total raw material cost [USD/Year]	28,046,300
Total product sales [USD/Year]	0
Total utility cost [USD/Year]	99,349.4
Desired rate of return [Percent/Year]	20
P.O. period [year]	0
Equipment cost [USD]	499,600
Total installed cost [USD]	1,063,700

Sensitivity analysis results shown in Table 10 revealed that TAC varied nearly linearly with IL price, ranging from 4.12 M USD/year at 50 USD/kg to 5.47 M USD/year at 400 USD/kg.^71^ Energy consumption (1.1 GJ/ton S), H_2_S conversion (≥99%), and sulfur recovery (≥99%) remained unaffected within this range, confirming that the ionic liquid cost is the significant parameter governing process economics.

**Table 10:** Sensitivity Analysis of the ILTM-Based Process

parameter	unit	lower bound	base case	upper bound
Total CAPEX	M USD	21.4	32.13	49.6
Total OPEX	M USD/year	1.28	1.53	1.89
TAC	M USD/year	4.12	4.803	5.47

In contrast, the elevated operational costs of the Claus processes are attributed to the inherent process complexities and energy demands of conventional technology.^57,59^ However, Aspen HYSYS lacks integrated modules for estimating the capital and operating costs associated with the Claus unit, the tail gas treatment section, and the incineration system.^14^ TAC further reinforces ILTM’s economic viability, with a value of 4.803 million MUSD compared to 8.291 MUSD for the Claus process, indicating superior overall cost-effectiveness. Figure 7 presents the CAPEX, OPEX, and TAC for both processes. These findings are consistent with the previous TEA studies reporting considerable savings in IL-based gas treatment processes.^32,33,57^ A detailed comparison of TEA of the ILTM and conventional Claus processes is presented in Table 11. The results indicate that the ILTM process offers clear economic and operational advantages. Specifically, ILTM exhibits significantly lower CAPEX than that of the Claus process and a reduced TAC. As a result, the TAC of ILTM is nearly half that of the Claus process, highlighting its superior cost efficiency. In addition to economic benefits, ILTM demonstrates substantially lower energy requirements, with an energy consumption of 1.1 GJ/t S, compared to 3.4 GJ/t of S for the Claus process. This improvement reflects enhanced process integration and reduced utility demand. Moreover, ILTM achieves higher H_2_S conversion (≥99%) and sulfur recovery efficiencies (≥99%), exceeding those of the Claus process, which range between 96 and 99% and 94 and 97%, respectively. From an environmental standpoint, ILTM shows nearly zero direct CO_2_ emissions, whereas the Claus process generates approximately 0.784 tons of CO_2_ per ton of sulfur produced. This further emphasizes the environmental advantages of the proposed ILTM technology. Although the high cost of [bmim]­[FeCl_4_] remains a constraint, ongoing advances in IL synthesis, recovery, and mixed-IL formulations are expected to enhance feasibility.^87^

**Figure 7:**
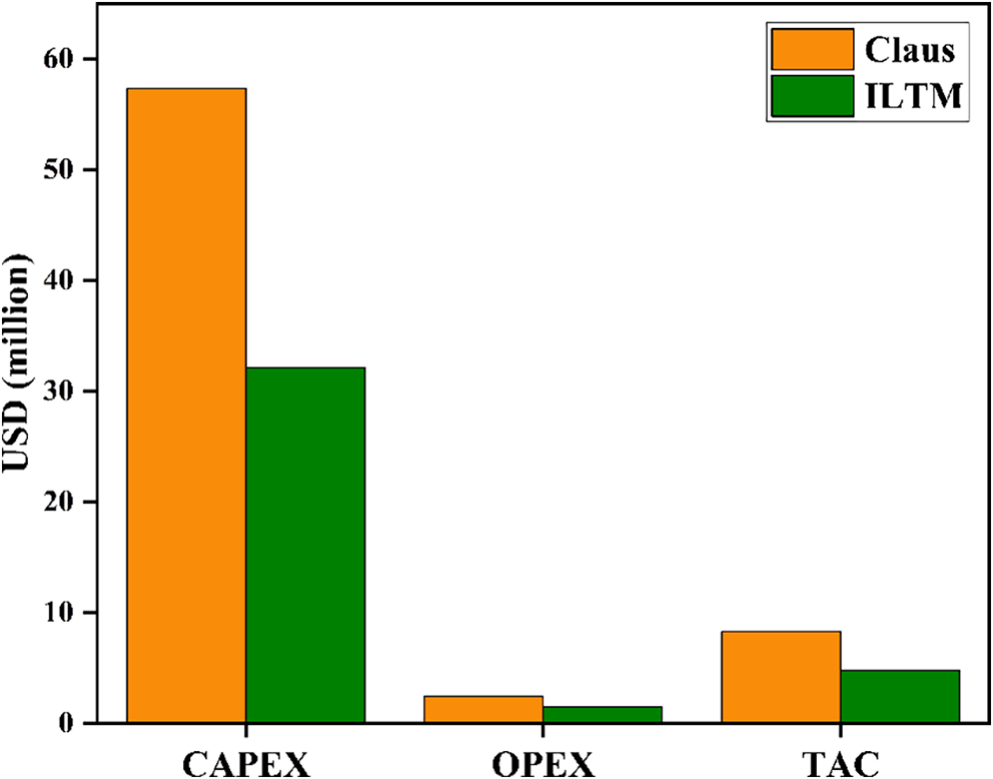
Claus and ILTM process CAPEX, OPEX, and TAC.

**Table 11:** Comparison of TEA Results

metric	unit	ILTM	Claus
Total CAPEX	M USD	32.13	57.35
Total OPEX	M USD/year	1.53	2.45
TAC	M USD/year	4.803	8.291
Energy consumption	GJ/t S	1.1	3.4
H_2_S conversion	%	≥99	96–99
Sulfur recovery	%	≥99	94–97
Direct CO_2_ emissions	CO_2_ t/t S	∼0	0.784

Overall, the ILTM-based process results demonstrate a technically robust and economically competitive alternative to the Claus process. It achieves near-complete sulfur recovery under mild conditions, requires simpler equipment, and offers lower CAPEX and OPEX. The process stability across temperature and pressure variations further enhances its industrial applications. However, future improvements in IL synthesis and regeneration efficiency will be crucial for scaled-up deployment. The ILTM process could serve as a sustainable and energy-efficient replacement for conventional sulfur recovery systems, aligning with global goals for cleaner and more cost-effective fuel processing.

## 4 Conclusions

The comparative technical and economic assessment of the ILTM and Claus processes for sulfur recovery under identical feed conditions demonstrates that the ILTM offers both higher process efficiency and superior cost performance. Technically, the ILTM process achieved a sulfur recovery efficiency of 99.68%, marginally surpassing the 99.56% obtained with the Claus process. This enhanced performance can be attributed to the high H_2_S solubility and absorption capacity of the ionic liquid [bmim]­[FeCl_4_], which enable effective H_2_S removal and conversion to elemental sulfur under mild operating conditions. Economically, ILTM requires 43.96% less capital expenditure than that of Claus, with CAPEX values of 32.13 million USD and 57.35 million USD, respectively. Operating expenses were also significantly lower for ILTM, at 1.53 million USD annually compared to the 2.45 million USD for Claus, reflecting reduced energy consumption and a simplified process configuration. These economic benefits are further reinforced by the lower TAC of ILTM, which was calculated as 4.803 million USD, compared with the 8.291 million USD for the Claus process, indicating superior lifecycle cost efficiency.

The operational advantages of ILTM ambient-temperature and low-pressure operation contribute to lower utility requirements and reduced equipment wear, while its potential for ionic liquid regeneration supports a long-term sustainability. Potential challenges related to ionic liquid degradation, water sensitivity, sulfur fouling, and scale-up effects were not explicitly modeled and may influence the real-world operation. Nevertheless, the high market cost of [bmim]­[FeCl_4_] remains a primary barrier to widespread adoption. Future work should focus on reducing solvent costs through advances in ionic liquid synthesis, recovery, and formulation. Experimental validation at the pilot scale is also required to confirm long-term operability, solvent stability, and sulfur handling under industrial conditions. Overall, the study establishes ILTM as a technically robust and economically viable alternative to the conventional Claus process, particularly in applications where energy efficiency and a reduced environmental impact are prioritized.

## Supplementary Material



## Notes

Effect of the rate constant (min^–1^) on H2S conversion, effect of the flow rate on H_2_S conversion, and effect of reactor temperature on H_2_S conversion (PDF)

## Notes

Declaration of Generative AI and AI-Assisted Technologies in the Manuscript Preparation Process: The author(s) used generative AI tools (such as ChatGPT by OpenAI) during the final writing process to improve the language, clarity, and readability of this manuscript. These tools were used only for writing and editing purposes. All ideas, scientific content, data interpretation, and conclusions are entirely the authors’ own work. The authors carefully reviewed and revised the final text and take full responsibility for its content.
