# Supplementary material for: Process Simulation and Economic Evaluation of the [bmim][FeCl4] Ionic Liquid for H2S Direct Conversion to Elemental Sulfur
Source: ACS Omega. 2026 Jul 8;11(28):41230–40. doi: 10.1021/acsomega.5c11851 (PMC13393047; doi:10.1021/acsomega.5c11851)
Supplement: Supplementary file 1 [file ao5c11851_si_001.pdf]

Supporting Information

Process Simulation and Economic Evaluation of [bmim][$FeCl_4$] Ionic Liquid for H_2S Direct Conversion to Elemental Sulfur

H. Abuzar Ahsan[1,2], *M. Azmi Bustam*[1,3], *H. Ali Murtaza*[1,2], *Abid Mehmood*[1], *Bawadi Abdullah*[1,2,]*

[1]Department of Chemical Engineering, Universiti Teknologi PETRONAS, Bandar Seri Iskandar, 32610 Perak, Malaysia

[2]Centre of Carbon Capture, Utilization and Storage (CCCUS), Institute of Sustainable Energy & Resources (ISER), Universiti Teknologi PETRONAS, Bandar Seri Iskandar, 32610 Perak, Malaysia

[3]Centre of Research in Ionic Liquids (CORIL), Institute of Sustainable Energy & Resources (ISER), Universiti Teknologi PETRONAS, Bandar Seri Iskandar, 32610 Perak, Malaysia

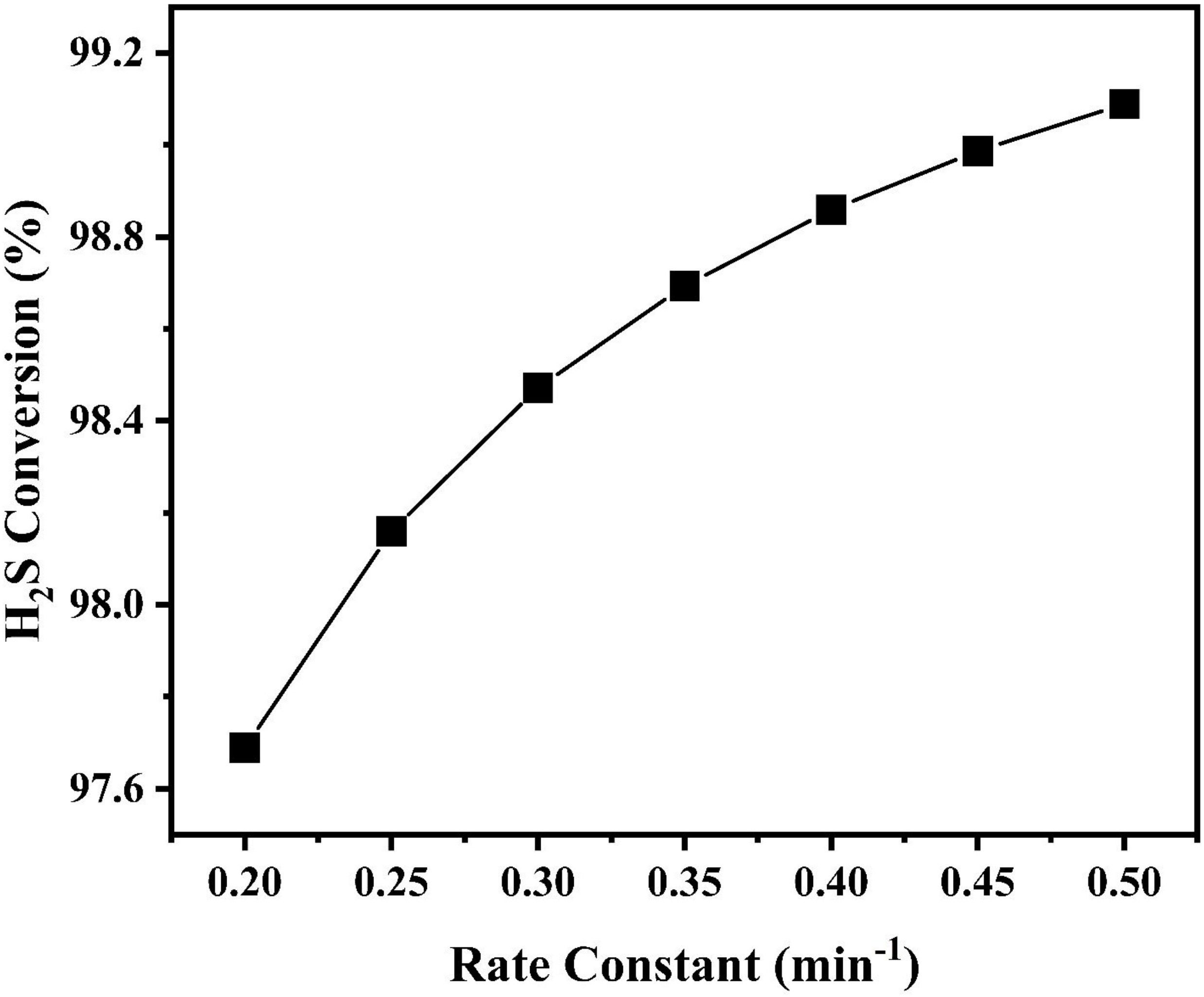

Figure S1. Effect of rate constant (min^{-1}) on H_2S conversion

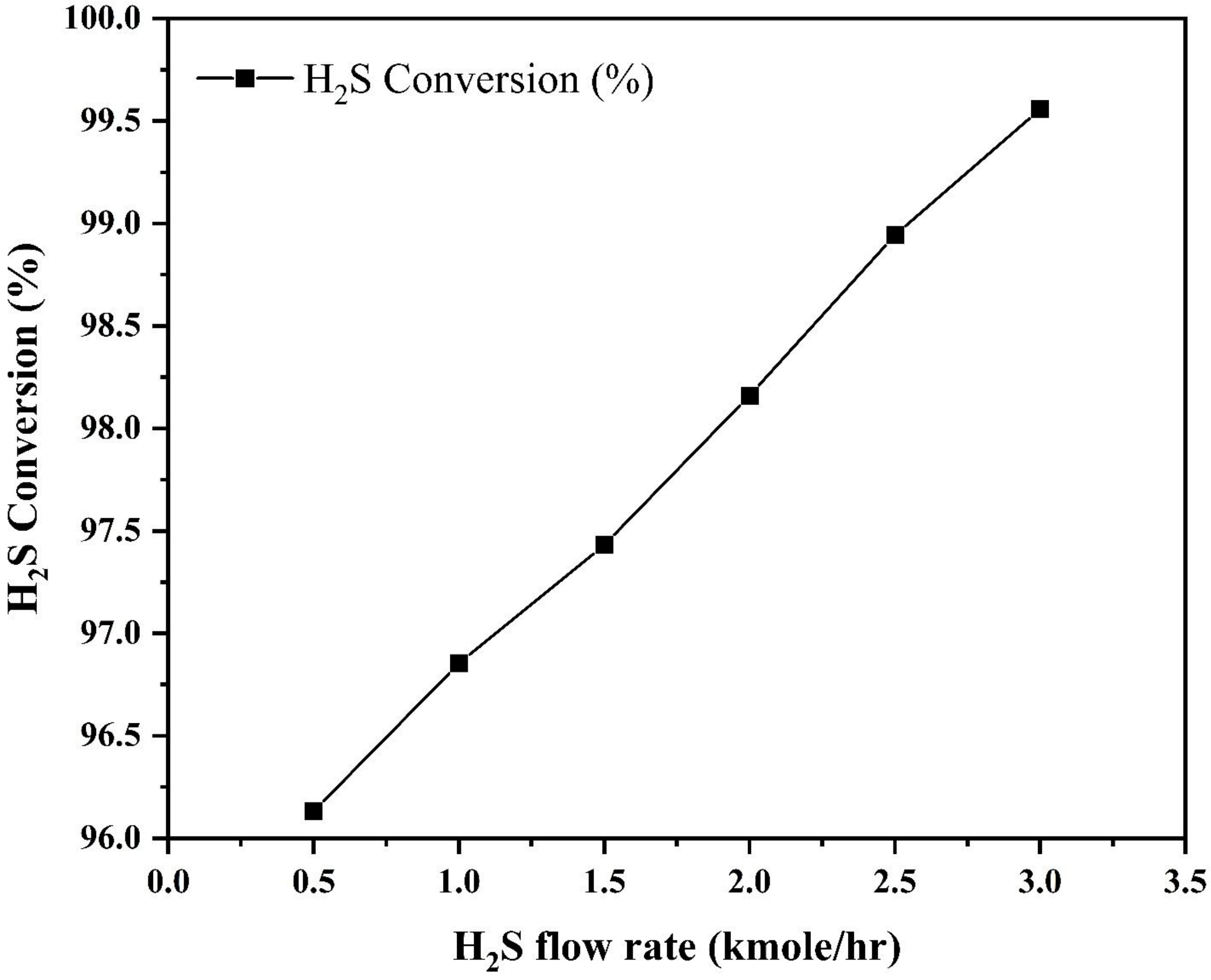

Figure S2. Effect of flow rate on H_2S conversion

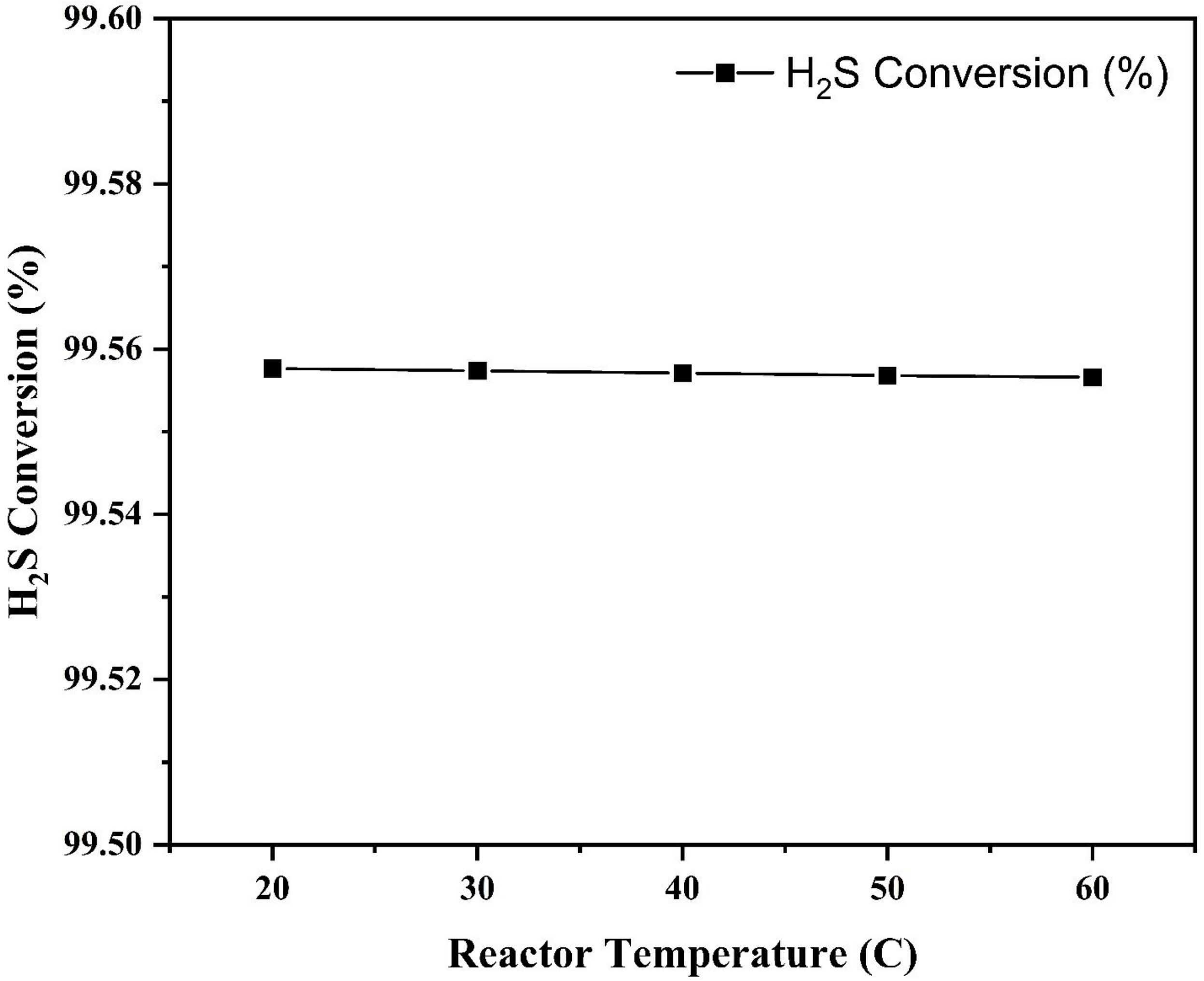

Figure S3. Effect of reactor temperature on H_2S conversion
